# Rethinking the planetary health diet: GBD data reveal a “sweet spot” for red and processed meat and longevity

**DOI:** 10.3389/fnut.2025.1736366

**Published:** 2026-02-09

**Authors:** David Keith Cundiff

**Affiliations:** Volunteer Collaborator with the Institute of Health Metrics and Evaluation, San Anselmo, CA, United States

**Keywords:** biointensive agriculture, global burden of disease data, meat consumption “sweet spot”, non-communicable diseases (NCDs), planetary health diet

## Introduction

The EAT-Lancet Commission Report is a sweeping global effort to define a “planetary health diet” (PHD) that nourishes humanity while remaining within ecological limits (1). Anchored in Stockholm, the initiative aspires to unite human and environmental health through a coherent nutritional framework.

While the vision is laudable, the scientific footing of its dietary targets—particularly those concerning animal-source foods—remains uncertain. The Commission relies heavily on the Theoretical Minimum Risk Exposure Level (TMREL) framework developed by the Institute for Health Metrics and Evaluation (IHME) (2). TMREL estimates the intake level of each food associated with the lowest disease risk and forms the backbone of the Global Burden of Disease (GBD) studies and, by extension, the PHD.

The idea of the TMREL predates the Institute of Health metrics and evaluation, which was founded in 2007. The concept of using a counter factual minimum risk level predates the IHME but the IHME formalized the TMREL term within the GBD's comparative risk assessment framework. Therefore, while the core idea is older, its formal use and publication by IHME as the TMREL within the detailed GBD study reports became standard practice in the mid-to-late 2010s.

TMREL values are derived primarily from meta-analyses of observational studies, largely conducted in high-income nations. Extrapolating these findings globally risks neglecting the nutritional realities of low- and middle-income populations still facing under nutrition and micronutrient deficiencies (3).

## The problem with TMREL

The wide fluctuations in TMREL estimates—ranging from zero to over 20 g/d—underscore the instability of current modeling approaches. Theoretical constructions may produce elegant visualizations but remain detached from real-world diversity. Ecological data, while imperfect, reflect how populations actually thrive or falter under varying dietary patterns.

Moreover, IHME's own GBD updates reveal striking instability in TMREL estimates for red meat: GBD TMREL.

Year | Red meat (g/d)

2019 | 22.5 | “Threshold for harm” (not TMREL)

2020 | 0 | Any intake deemed harmful

2022 | 0 (95% UI: 0–200) | Extremely wide uncertainty

Such volatility suggests model instability rather than empirical consistency. The EAT-Lancet Commission, whose Planetary Health Dietary recommendations generally mirror these IHME TMREL estimates—red meat 15 g/d (range 0–28) and processed meat 0 g/d—rests on an uncertain foundation.

## Methods

In 2023, statistician Chunyi Wu, PhD, and I compared the EAT-Lancet Planetary Health Diet (PHD) with Global Burden of Disease (GBD)–derived mortality and dietary data (4). The full GBD data analysis methodology underlying the tables and results in this report is detailed in that publication. All associated Data and Statistical Analysis System (SAS) code is publicly available on Mendeley depository (5).

We obtained the IHME GBD data as volunteer collaborators in 2018, receiving the raw GBD 2017 dataset in R format. Readers may download the analysis database (termed: wtedCVDRfsCov2017), along with (1) SAS code to import the data, (2) SAS code to format the database for analyses, and (3) multiple SAS programs for evaluating associations between health outcomes—such as non-communicable disease (NCD) early deaths (ages 15–69)—and approximately 20 food variables, physical activity, smoking, air pollution, and other risk factors.

Readers are encouraged to become IHME volunteer collaborators and obtain GBD 2023 raw data to further extend the analyses that we have done.

## Results

### A GBD data–driven alternative

The dietary intakes and premature NCD mortality (ages 15–69) across 1,000 cohorts (1 million people per cohort) representing the world's longest-lived 1 billion people are presented in Table 1. Table 2 has 1,014 cohorts with NCD deaths/100k/year m/f ages 15–69 > 1,070 and Sociodemographic index (SDI) > 0.67.

**Table 1:** GBD data from 1990–2017 representing the world's longest living people.

**Label**	***N***	**Mean**	**Std dev**	**Minimum**	**Maximum**
NCD deaths/100k/year m/f ages 15–69	1,000	925.2	113.11	634.58	1,070
Year | Red meat (g/d)	1,000	28.21	16.77	4.14	70.57
Processed meat g/d	1,000	6.31	5.98	0.44	34.07
Fish g/d	1,000	38.24	72.57	2.08	289.34
Milk g/d	1,000	108	64.45	29.19	293.64
Alcohol g/d	1,000	14.75	9.41	0.61	42.39
Sugar sweetened beverages g/d	1,000	87.59	63.99	18.23	367.9
Fruits g/d	1,000	106.3	30.97	36.02	189.52
Vegetables g/d	1,000	163.2	73.94	14.59	351.92
Nuts and seeds g/d	1,000	2.53	2.01	0.04	7.83
Whole grains g/d	1,000	21.67	13.93	0.70	71.49
Legumes g/d	1,000	36.07	20.01	2.17	96.94
BMI kg/M^2^ m/f	1,000	23.76	1.42	20.20	28.22
LDLc mmol/L m/f	1,000	2.74	0.29	1.69	3.24
SBP mm Hg m/f	1,000	134.1	3.76	125.19	143.43
Type 2 diabetes deaths m/f/100k/year	1,000	12.59	13.27	0.75	69.45
CVD deaths m/f/100k/year	1,000	274.7	109.32	135.46	551.23
Common cancer deaths m/f/100k/year	1,000	215.5	110.07	66.07	457.05
Socio-demographic index (0–1)	1,000	0.72	0.14	0.35	0.89
Physical activity METs m/f	1,000	3,389	1,019	1,609	7,607

**Table 2:** Cohorts of people with Socio-demographic index (SDI) > 0.67 and are not in Table 1.

**Label**	***N***	**Mean**	**Std dev**	**Minimum**	**Maximum**
NCD deaths/100k/year m/f (ages 15–69)	1,014	1,504	427	1,070	2,754
Red meat g/d	1,014	37.23	14.34	7.61	81.08
Processed meat g/d	1,014	10.01	8.16	0.25	35.09
Fish g/d	1,014	8.41	3.04	1.43	20.21
Milk g/d	1,014	130.9	53.58	10.76	264.56
Alcohol g/d	1,014	18.00	11.17	0.95	61.40
Sugar sweetened beverages g/d	1,014	70.36	42.24	18.75	339.96
Fruits g/d	1,014	91.08	32.97	26.95	267.25
Vegetables g/d	1,014	167.9	54.80	38.91	467.96
Nuts and seeds g/d	1,014	2.62	1.90	0.01	17.82
Whole grains g/d	1,014	20.85	13.04	0.69	75.09
Legumes g/d	1,014	20.51	1 15.00	0.46	95.87
BMI kg/M^2^ m/f	1,014	25.00	1.49	19.67	29.29
LDLc mmol/L m/f	1,014	2.84	0.23	1.74	3.17
SBP mm Hg m/f	1,014	134.7	5.92	124.18	145.39
Type 2 diabetes deaths m/f/100k/year	1,014	13.61	17.28	0.63	261.23
CVD deaths m/f deaths/100k/year	1,014	567.4	363.3	224.32	1,258.00
Common cancer deaths m/f/100k/year	1,014	315.5	140.5	108.12	710.47
Socio-demographic index (0–1)	1,014	0.80	0.06	0.67	0.90
Physical activity METs m/f	1,014	4,341	1,212	1,952	6,956

Compared to Table 1, Table 2 cohorts consumed 37% more red and processed meat, only 22% of the fish intake, and 57% of the legume consumption. Additionally, the average BMI of Table 2 cohorts was 1.24 units higher than that of Table 1 cohorts.

### The “sweet spot” for meat and longevity

Synthesizing these findings, the optimal longevity range in Table 1 corresponds to roughly 34.5 g/d of combined red and processed meat—approximately 1.7 times the current meat consumption global percapita g/d average. This suggests that humanity may benefit from about 70% more, not less, animal-source food, particularly among the lower SDI of three-fourths of the human population (Table 3).

**Table 3:** About 5.8 billion people from developing countries, SDI < 0.67, and not in Table 1.

**Label**	***N***	**Mean**	**Std dev**	**Minimum**	**Maximum**
NCD deaths/100k/year m/f (ages15–69)	5,832	1,501	298	1,072	3,521
Red meat g/d	5,832	11.93	11.02	1.10	66.16
Processed meat g/d	5,832	0.84	0.64	0.10	13.53
Fish g/d	5,832	2.48	1.95	0.31	21.67
Milk g/d	5,832	26.09	21.02	2.11	207.51
Alcohol g/d	5,832	9.92	6.46	0.82	48.62
Sugar sweetened beverages g/d	5,832	73.10	30.11	42.81	367.12
Fruits g/d	5,832	56.09	32.48	5.97	268.98
Vegetables g/d	5,832	107.91	60.06	16.66	364.22
Nuts and seeds g/d	5,832	1.07	0.95	0.01	8.64
Whole grains g/d	5,832	25.07	13.21	0.49	101.77
Legumes g/d	5,832	40.33	23.60	0.37	140.07
BMI kg/M^2^ m/f	5,832	20.86	1.66	18.28	27.74
LDLc mmol/L m/f	5,832	2.20	0.31	1.34	3.04
SBP mm Hg m/f	5,832	133.75	3.78	124.71	147.05
Type 2 diabetes deaths m/f/100k/year	5,832	19.02	15.44	0.89	269.67
CVD deaths m/f deaths/100k/year	5,832	585.70	205.61	213.31	1,727.00
Common cancer deaths m/f/100k/year	5,832	205.82	120.29	71.67	875.42
Socio-demographic index (0–1)	5,832	0.47	0.12	0.11	0.67
Physical activity METs m/f	5,832	5,005	1,295	1,666	7,669

US data are revealing. Only Alaska, Colorado, Hawaii, Washington, and Wyoming (Totaling 26 million US residents out of 336 million) were included in Table 1. The rest were in Table 2. Washington, DC had the highest US mortality rate—1,870.5 NCD deaths m/f/100k/year. Mississippi was second—1,500 NCD deaths m/f/100k/year. Average meat consumption for US residents was 68.1 g/d, compared with 47.2 g/d mean meat intake in Table 2 and 34.5 g/d in Table 1.

## Discussion

It should be noted that ecological associations cannot infer individual-level causality. While empirical GBD data show that the sweet spot of combined processed meat and red meat consumption is roughly 34 g per day, individual level causality Is not inferred and would present an ecological fallacy (e.g., “more meat = better survival or less meat equals better survival”).

LDL-c mmol/L levels of Tables 1 and 2 were very similar and far higher than in Table 3. LDL-c showed limited sensitivity at ecological scale, indicating possible scale-mismatch when informing global dietary TMREL targets. Patterns and relationships observed in nature can change dramatically depending on the scale at which you look at them.

Potential confounders inherent to ecological comparisons—such as food system availability, healthcare access, infectious disease burden, environmental exposures, and total energy intake—are competing explanations for mortality differences, particularly across SDI strata.

In all three Tables, mean SBP mm Hg differed little while NCDs m/f/100k/year increased over 60% in Table 2 and Table 3, possibly indicating that intakes of foods were the primary influences on longevity and SBP secondary in determining NCD early deaths. Meanwhile CVD deaths in Table 2 (585.70 CVD deaths/100K/year) and Table 3 (567.35 CVD deaths/100K/year) far surpassed 274.65 CVD deaths/100K/year in Table 1. This implies that food profiles, particularly meat, contribute strongly, perhaps by different mechanisms, to early CVD mortality. Along the same line, Table 2 shows that early cancer and CVD mortality were both high and associated with high red and processed meat.

These results parallel the findings of You et al. (6) who analyzed 175 populations using FAO and WHO data and found that higher total meat intake correlated with longer life expectancy, even after adjusting for caloric intake, urbanization, and education. Together, these data suggest that moderate meat consumption contributes to optimal health outcomes, particularly where nutrient deficiencies persist as in Table 3. Based on these data, people with diets fitting in with Table 2 data might consider reducing or eliminating meat consumption while increasing fish and lentils. Conversely, people with low meat intake in Table 3 could try to markedly increase overall intake of animal-based as well as plant-based food.

The dietary contrast between high-SDI cohorts (Table 2) and the longest-lived cohorts (Table 1) —especially the much lower fish and legume intake in Table 2 despite higher meat exposure—deserves deeper mechanistic discussion to support the hypothesis of a composite dietary profile effect rather than a single food driver.

Notably, the average metabolic parameters in Tables 1, 2 were not significantly different except for BMI and LDL-c in Table 3: BMI kg/M^2^ (23.76 BMI kg/M^2^ m/f Table 1 vs. 25.0 BMI kg/M^2^ m/f Table 2 vs. 20.86 in Table 3), LDL cholesterol mmol/L (LDL cholesterol: 2.74 mmol/L Table 1 vs. 2.84 mmol/L Table 2 vs. 2.20 in Table 3), systolic blood pressure mm HG (SBP: 134.1 mm Hg Table 1 vs. 134.7 mm Hg Table 2 and 133.75 mm Hg in Table 3), and type 2 diabetes deaths (12.59/100K/year in Table 1 vs. 13.61/100K/year in Table 2 vs. 19.02/100K/year in Table 3).

To monitor intake of food to decrease BMI and/or to decrease risk of cancer and CVD, apps that may help in accurately following food intake include Levels (7) and Cronometer (8).

## The ecological impact of agriculture in creating a healthful diet

To craft credible global dietary policy, models must integrate data from diverse regions and income levels. For much of humanity, animal-source foods remain vital for nutrient adequacy, child development, and disease resilience. A planetary diet that disregards this biological and social reality risks deepening, rather than narrowing, global health inequities.

The consensus across major international bodies and peer-reviewed literature is that the expansion of conventional, high-impact animal agriculture (especially confined animal feeding operations (CAFOs)) is a leading global driver of negative environmental change (9).

For example, livestock in African countries contribute to about 10% of enteric methane emissions from dairy cattle worldwide despite producing only 3.9% of the world's milk. Livestock in Sub-Saharan Africa (SSA) also cause extensive land degradation with 48% of rangelands in SSA degraded due to overgrazing. Strategies for sustainable intensification of livestock such as improving quality of feed, wholistic grazing as promoted by Alan Savory (10), land rehabilitation, introduction of improved forages and silvopastoral systems, and improvement of herd genetics can reduce both total emissions and emission intensity while improving productivity (11).

According to the Food and Agriculture Organization of the UN (12), the underlying causes of climate change, such as reliance on conventional agriculture grown with chemical fertilizers, herbicides, and pesticides for 98% of food for humanity, must be addressed by shifting from conventional agriculture to growing food with organic, regenerative methods. Poor farmers in developing countries especially benefit from farming biointensively with hand tools, growing their fertilizers, saving seeds, close spacing of plants to save on water, weeding, fossil fuels, and land (13). If scaled globally with communities living in ecovillages with few or no fossil fuel requiring vehicles, biointensive farming could dramatically reduce greenhouse gas emissions.

## Conclusion

The EAT-Lancet Commission has sparked an essential conversation linking diet, health, and planetary boundaries. Yet inspiration must be grounded in empirical coherence. The volatility of TMREL estimates and their limited alignment with global dietary data highlight the need for recalibration. IHME's own GBD 1990–2017 data—contrary to TMREL modeling—offers consistent, population-level evidence capable of identifying real-world nutritional “sweet spots”—e.g., red meat and processed meat in Table 1 vs. Tables 2, 3. Empirically derived, globally representative dietary thresholds can align nutritional guidance with both human vitality and planetary stewardship. Future planetary health research should embrace this empirical turn—toward nutritionally adequate, culturally diverse, and environmentally balanced diets that sustain both people and planet. The EAT-Lancet Commission has sparked an essential conversation linking diet, health, and planetary boundaries rally diverse, and environmentally balanced diets that sustain both people and planet.

## Data Availability

Publicly available datasets were analyzed in this study. This data can be found here: https://data.mendeley.com/datasets/64gv2ffx72/1.
